# Identification and characterization of gonadotropin-releasing hormone (GnRH) in Zhikong scallop *Chlamys farreri* during gonadal development

**DOI:** 10.3389/fphys.2023.1180725

**Published:** 2023-05-31

**Authors:** Juyan Tang, Mengqiang Yuan, Jia Wang, Qianqian Li, Baoyu Huang, Lei Wei, Yaqiong Liu, Yijing Han, Xuekai Zhang, Xiaona Wang, Meiwei Zhang, Xiaotong Wang

**Affiliations:** ^1^ School of Agriculture, Ludong University, Yantai, China; ^2^ College of Animal Science and Technology, Northwest A&F University, Xianyang, China

**Keywords:** *Chlamys farreri*, GnRH, nerve ganglia, gonadal development, *in situ* hybridization

## Abstract

Gonadotropin-releasing hormone (GnRH) controls synthesis of sex steroid hormones through hypothalamic-pituitary-gonadal (HPG) axis in vertebrates. But in mollusks, research on neuroendocrine control of gonadal function, such as the function of GnRH during gonadal development is limited. In this study, we investigated the morphology and structure of the nerve ganglia of Zhikong scallop *Chlamys farreri* by physiological and histological observations. We also cloned the ORF and studied the expression patterns of *GnRH* in the scallop. Tissue expression analysis showed that *GnRH* was highly expressed in parietovisceral ganglion (PVG). The *in situ* hybridization result further confirmed that *GnRH* mRNA only distributed in some good-sized neurons in the posterior lobe (PL) and some pint-sized neurons in the lateral lobe (LL). In addition, by examining the expression of *GnRH* during gonadal development in ganglia, we found *GnRH* displayed higher expression in the female scallops, and showed significant high expression at the growing stage of female scallops in PVG. This study would contribute to gaining insight into the mechanism underlying reproduction regulation by GnRH in the scallop and help to provide a better understanding of reproductive neuroendocrine in mollusks.

## 1 Introduction

Gonadotropin-releasing hormone (GnRH) is the major neurohormone in hypothalamic–pituitary–gonadal (HPG) axis, which plays a key role in sexual development and reproduction regulation in vertebrates (Bliss et al., 2010). GnRH is released from hypothalamus and received by the receptor for releasing gonadotropins including luteinizing hormone and follicle-stimulating hormone in the pituitary (Jin and Yang, 2014). These hormones are then transported to the gonads to stimulate the synthesis of sex steroid hormones, and then modulate reproductive process (Huggard-Nelson et al., 2002; Schulster et al., 2016). In invertebrates, with an absence of complex brain tissues such as hypothalamus and pituitary, GnRH also be found in nervous system and may play an important role of reproductive function (Tinikul et al., 2014; Sukhan et al., 2021; Ojima et al., 2022).

Mollusks are one of the most abundant and biologically diverse groups in the animal kingdom. Although they do not possess the complex brain structure as vertebrates, they have various kinds of nerve ganglia as central nervous system (CNS). The neurohormones are synthesized in nerve ganglia and released into the haemolymph via a network of neurohemal organs to regulate growth, immune, reproduction and other physiological functions (Zhang et al., 2018; Li et al., 2019; Kotsyuba and Dyachuk, 2022). Although there is no “HPG axis” in mollusks, GnRH has been identified in various mollusks (Zhang et al., 2008; Bigot et al., 2012; Nagasawa et al., 2015; Kim et al., 2017; Rosa-Casillas et al., 2021). Molluscan GnRH was suggested to be associated with reproduction (Morishita et al., 2010; Osada and Treen, 2013; Ding et al., 2021; Murata et al., 2021).

The Zhikong scallop, *Chlamys farreri* is an important maricultural bivalve in China and Japan. Due to its commercial value, the reproduction of *C. farreri* has received a lot of research focus. Previously, vertebrate-type steroids and steroidogenesis genes have been reported in *C. farreri* and seem to regulate reproductive events (Qin et al., 2012; Liu et al., 2014a; Zheng et al., 2014). But the study on reproductive regulation of neuroendocrine in *C. farreri*, especially the potential reproductive function of GnRH, is still blank until now. In order to illustrate how the GnRH regulate gonadal development, we investigated the CNS and gonads of Zhikong scallop and studied its tissue and spatiotemporal expression. We suggested that GnRH may display an important role at the growing stage of female Zhikong scallops. This study will contribute to a better understanding of reproductive neuroendocrine in mollusks.

## 2 Materials and methods

### 2.1 Sample collection and histology

One-year-old scallops were obtained from Yantai, Shandong Province, China. The scallops were acclimated at 16°C in aerated seawater (30‰) for 1 week, and were fed with *Spirulina platensis* powder. After acclimation, nerve ganglia, gonadal tissues, peripheral tissues (mantle, gill, kidney, digestive gland and adductor muscle) were dissected, immediately frozen in liquid nitrogen and stored at −80°C before use. The gonadal development stages were distinguished by examining gonadal morphology and by histological observations. The structure of neural ganglion system was also investigated by histological observation.

### 2.2 RNA extraction and first-strand cDNA synthesis

Total RNA was extracted using Tiangen RNA Extraction Kit (Tiangen Biotech, China). RNA concentration and purity were determined using a Nanovue Plus spectrophotometer (GE Healthcare, NJ, United States), and RNA integrity was verified by agarose gel electrophoresis. First-strand cDNA synthesis was performed using the Evo MMLV Plus cDNA Synthesis Kit (Accurate Biology, China). The reverse transcription reaction conditions were as follows: 30 min at 42°C and termination by heating at 95°C for 5 min.

### 2.3 Cloning of *GnRH* ORF and sequences analyses

Using the *GnRH* gene sequences of other scallops *Mizuhopecten (Patinopecten) yessoensis* (XM_021491876.1), *Pecten maximus* (XM_033891046.1) and *Argopecten irradians* (Li et al., 2020) through alignment, we designed primers (Supplementary Table S1) to amplify *GnRH* gene. After purification with the SanPrep Column DNA Gel Extraction Kit (Sangon Biotech, China), the PCR product was ligated into the pEASY-T1 vector (TransGen Biotech, China). Then, the T-vector was transformed into competent *E. coli* cells. The positive clones were selected and sent to Personal BioTechnology (Shanghai, China) for sequencing.

### 2.4 Gene identification and phylogenetic analyses

After sequencing, the amino acid sequence was deduced. The function domains were predicted using the Simple Modular Architecture Research Tool (SMART) (http://smart.emblheidelberg.de). The GnRH family protein sequences of different species were obtained from the NCBI (http://www.ncbi.nlm.nih.gov/guide/proteins/) and MolluscDB (http://mgbase.qnlm.ac/page/download/download) (Liu et al., 2021) database. Multiple sequence alignments were performed with ClustalW (Thompson et al., 1994), and the results were annotated with GeneDoc. A phylogenetic tree was constructed using the neighbor-joining method with 1,000 bootstrap replicates using the MEGA7 (Kumar et al., 2016).

### 2.5 Reverse transcription quantitative PCR (RT-qPCR)

Gene-specific primers were designed using Primer Premier 5.0 (Supplementary Table S1). RT-qPCR was conducted using SYBR Premix Ex Taq II (TaKaRa, Japan) and a Bio-Rad CFX Connect PCR instrument with the following program: 94°C for 10°min, followed by 40 cycles of 94°C for 15°s and 60°C for 1 min EF1A (elongation factor 1-alpha) was used as an endogenous control for the normalization of gene expression. The relative expression level of each gene was calculated using the 2^−ΔΔCt^ method. All data were analyzed using SPSS 21.0 (IBM Corp, Armonk, United States). *p*-values lower than 0.05 were considered statistically significant.

### 2.6 Tissue *in situ* hybridization

The cDNA fragments were amplified with the GnRH_ISH primers (Supplementary Table S1). The template for *in vitro* transcription was conducted using the primers containing T7/SP6 promoter sequence. The anti-sense and sense probes were *in vitro* transcribed with a DIG RNA Labeling Kit (SP6/T7) (Roche, Mannheim, Germany). Sections of the PVG were serially rehydrated in PBST and digested with 2 mg/mL proteinase K at 37°C for 13 min. After pre-hybridization at 60°C for 4 h, hybridization was performed with 1 mg/mL denatured RNA probe in hybridization buffer at 60°C for 16 h. After hybridization, the probes were washed away, and antibody incubation was performed in a fresh solution of anti-digoxigenin-AP Fab fragments (Roche, Mannheim, Germany) coupled with blocking buffer (diluted 1:2000) at 4°C for 14 h. After extensive washing with maleic acid buffer, sections were incubated with NBT/BCIP substrate solution in the darkness and counterstained with 1% neutral red solution.

## 3 Results

### 3.1 Physiological and histological observation of nerve ganglion system

**FIGURE 1:**
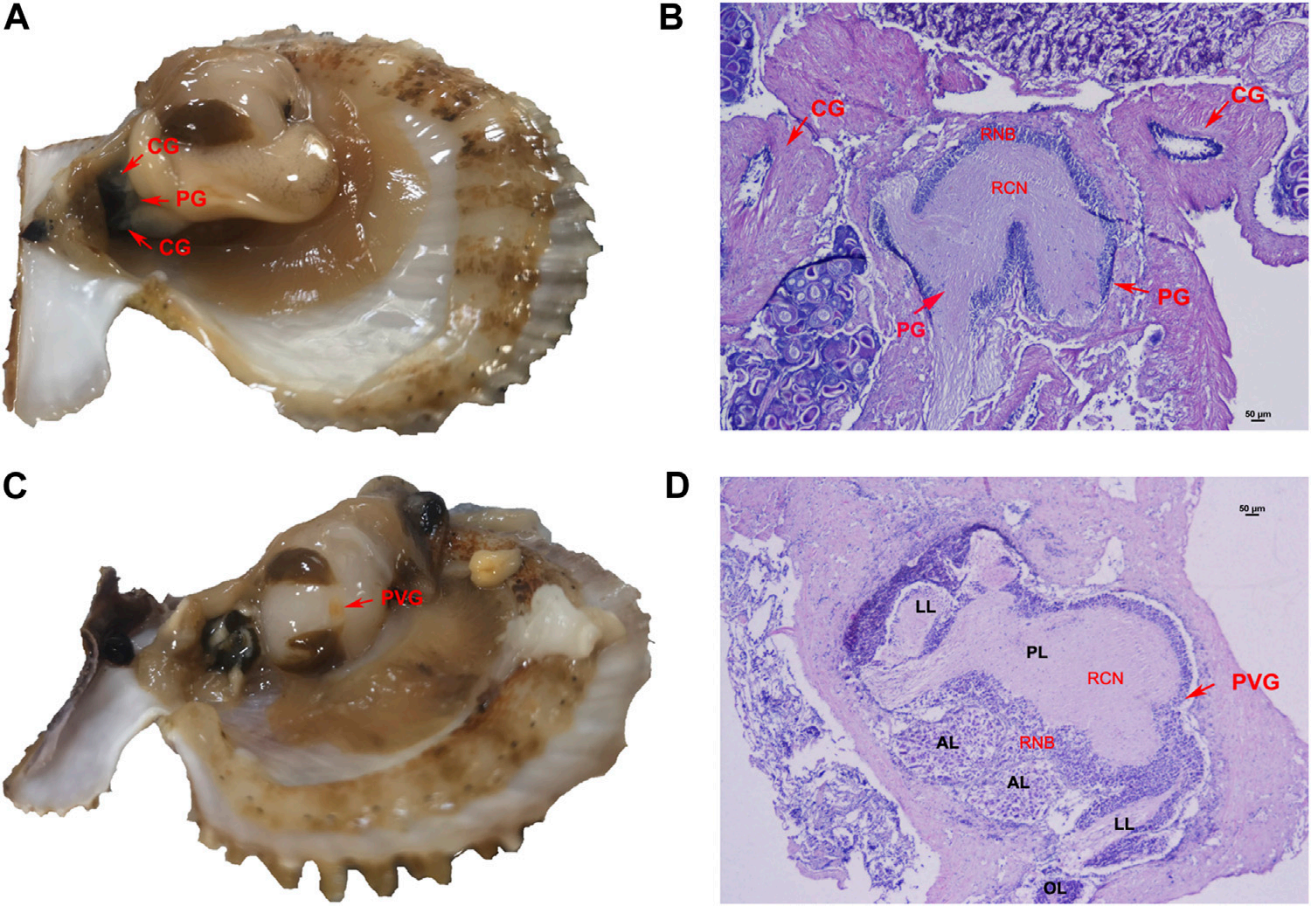
Nerve ganglion system of *C. farreri*. **(A)** morphological and **(B)** histological structure of CG and PG. **(C)** morphological and **(D)** histological observation of PVG. *Abbreviations*: RCN, region of central neuropile; RNB, region of neuron-cell bodies; AL, anterior lobe; LL, lateral lobe; PL, posterior lobe; OL, osphradium lobe.

The neural ganglion system of *C. farreri* consisted of one pair of separated cerebral ganglia (CG), one pair of connected pedal ganglia (PG) and one parietovisceral ganglion (PVG). By the physiological observation, the CG and PG were located next to each other between the gonad and the hepatopancreas (Figure 1A). By HE staining and tissue section observation, ganglia consisted of a region of neuron-cell bodies (RNB) and a region of central neuropile (RCN). The nerve fibers were distributed in the central part of ganglia or along nerve cords, where were colored lighter. Neuronal cell bodies in ganglia section were mainly distributed in the outboard surface where were colored deeply. The majority of neurons in the CG were the good-sized (>15 μm), and the number of neurons at pint size (4–5 μm) were more than that of those in good size in PG (Figure 1B). The PVG was above the middle region of adduct muscle, where it connected with the gonad (Figure 1C). The structure of PVG was comparatively complex (Figure 1D), including two anterior lobes (AL), one posterior lobe (PL), two lateral lobes (LL) and two osphradium lobes (OL). There were more good-sized neuron cells in AL and PL, and only pint-sized cells in LL and OL. Different lobes could be distinguished by the tissue morphology and the neuron size.

### 3.2 Sequence characterization and phylogenetic analysis

**FIGURE 2:**
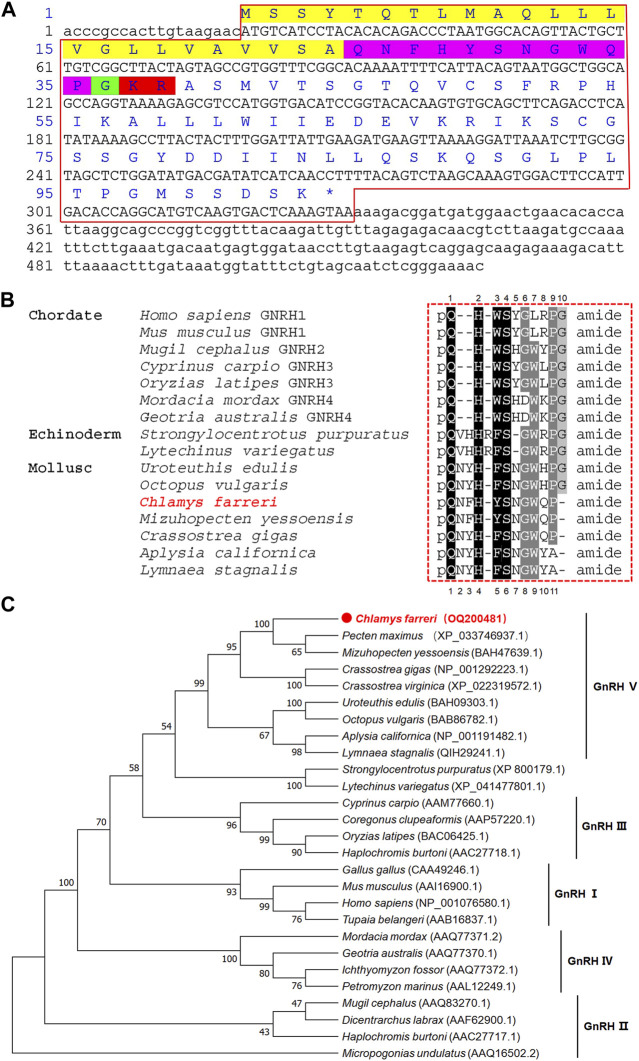
Sequence and phylogenetic analysis of *GnRH*. **(A)** Sequence of Zhikong scallop GnRH. Yellow, signal peptide; purple pink, GnRH peptide; green, C-terminal glycine; red, cleavage site. **(B)** Alignment of GnRH peptides among vertebrates, echinoderms and mollusks. **(C)** Neighbor-joining phylogenetic tree of *GnRH*.

The cDNA sequence of *GnRH* was obtained from nerve ganglia, and deposited in GenBank with an accession number of OQ200481. The partial-length cDNA consisted of 525-bp with a 5′-UTR of 19-bp, a 3′-UTR of 194-bp, and an open reading frame (ORF) of 312-bp (Figure 2A). The ORF encoded a precursor of 103 residues, including a 24-residue signal peptide, a conserved 11 residue GnRH peptide and a GKR sequence serving as a combined amidation and cleavage site. The deduced mature GnRH peptide was aligned with representative vertebrate GnRH1, GnRH2, GnRH3 and GnRH4 sequences along with a variety of echinodermal and molluscan GnRH members. Mature Cf-GnRH exhibits a high degree of identity with other molluscan GnRHs as well as some relatedness with vertebrate and echinodermal GnRHs (Figure 2B). The phylogenetic tree of GnRH precursor sequences showed that vertebrate GnRHs can be classified into four groups: GnRH-I (hypothalamic/preoptic forms of GnRH), GnRH-II (mesencephalic form of GnRH), fish-specific GnRH-III and lamprey-specific GnRH-IV, as previously reported (Roch et al., 2014). The sequence of Cf-GnRH clustered mainly with invertebrates, especially classified as a member of molluscan GnRH-V (Figure 2C).

### 3.3 Tissue distribution and spatial expression of *GnRH*

**FIGURE 3:**
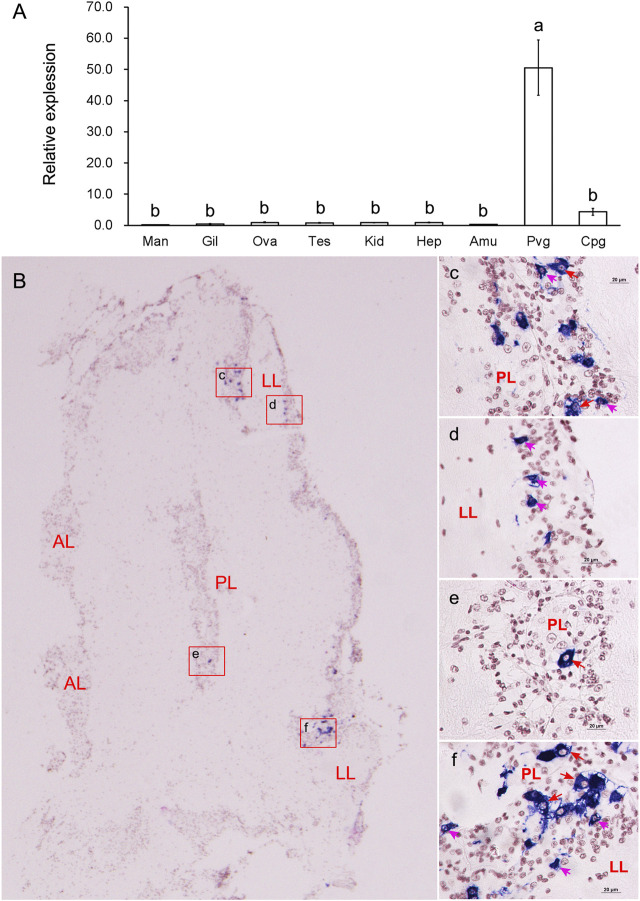
Tissue expressions and spatial localization of *GnRH*. **(A)** Relative expressions in different tissues. *Abbreviations*: Man, mantle; Gil, gill; Ova, ovary; Tes, testis; Kid, kidney, Hep, hepatopancreas; Amu, adduct muscle; Pvg, parietovisceral ganglion; Cpg, cerebral and pedal ganglia. **(B)** Localization of GnRH mRNA in PVG. **(C-F)**: The enlarged areas in the red boxes corresponding to panel **(B)**. Positive signals with an antisense probe are indicated in blue, and the cell nuclei are dyed red. Red arrows indicate good-sized neurons; pink arrows indicate pint-sized neurons.

Relative expression analysis revealed that the mRNA expression of *GnRH* was mainly detected in the nerve ganglia. It was significantly (*p* < 0.05) higher in the PVG than in any other examined tissues (Figure 3A). We further investigated the spatial expression in PVG using *in situ* hybridization (Figure 3B). Strong hybridization signals were found in the cytoplasm of some good-sized neurons (15–20 μm) in posterior lobe (PL) (Figures 3C, E, F) and some pint-sized neurons (<15 μm) in lateral lobe (LL) (Figures 3D, F). The mRNA was only detected in a few dispersed neurons, but most neurons did not have any signal distribution.

### 3.4 Temporal expression of *GnRH* in neural ganglia during gonadal development

**FIGURE 4:**
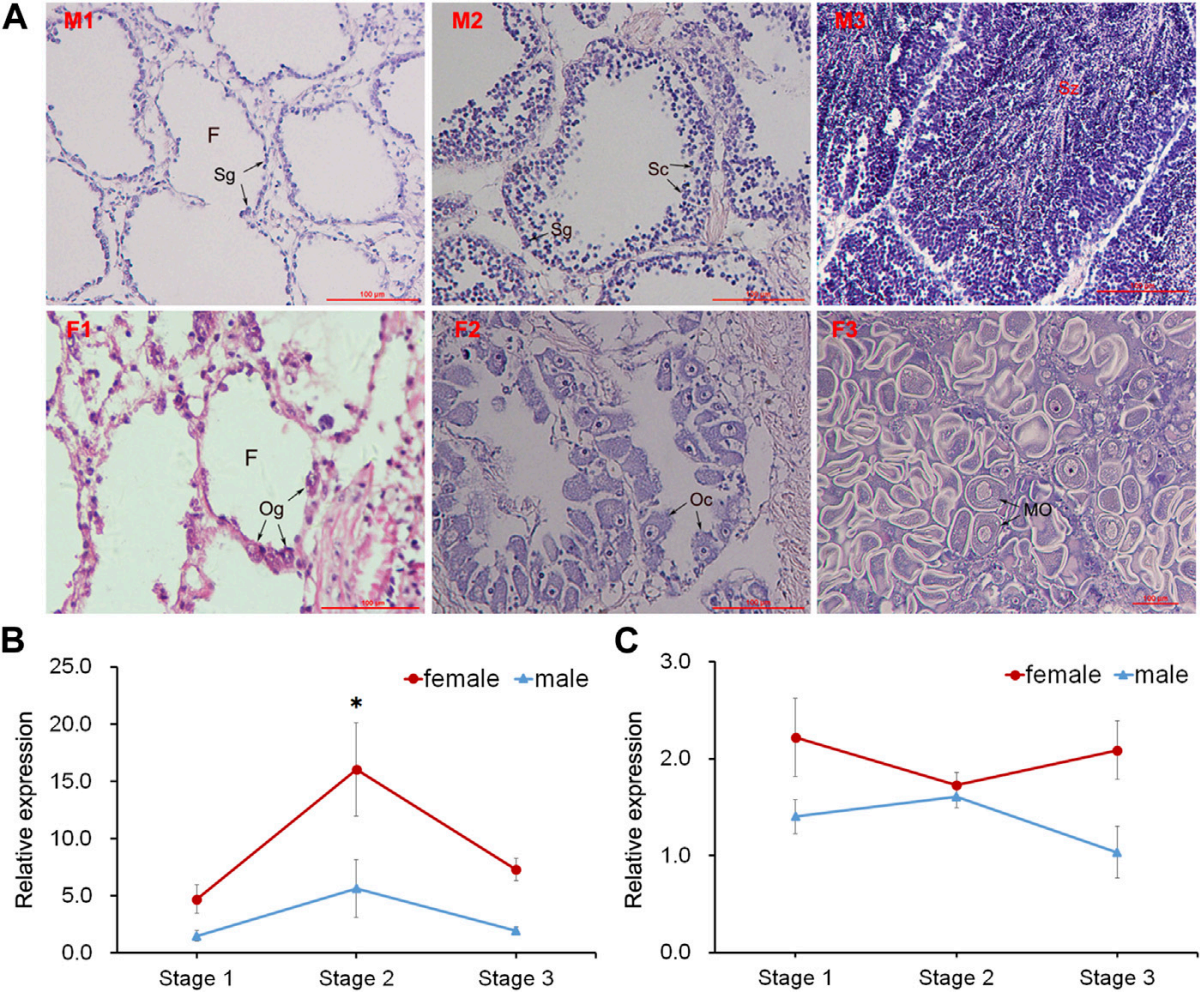
Histological observation of gonads and temporal expression of *GnRH* in ganglia. **(A)** Sections of testis and ovary during different gonadal development. *Abbreviations*: M, testis; F, ovary; 1, proliferative stage; 2, growing stage; 3, mature stage. F, follicle; Sg, spermatogonium; Sc, spermatocyte; Sz, spermatozoon; Og, oogonium; Oc, oocyte; MO, mature oocyte. Relative expressions of *GnRH* in **(B)** PVG and **(C)** CPG.

Three different stages (proliferative stage, growing stage and mature stage) of testis and ovary were distinguished by histological observation (Figure 4A). The *GnRH* in nerve ganglia displayed higher expression in the female scallops than in the male individuals. During the gonadal development, *GnRH* displayed significant high expression at the growing stage of female scallops in PVG (Figure 4B). But the expression of *GnRH* in CPG didn’t show any significant change during the three stages in female and male scallops (Figure 4C).

## 4 Discussion

In the present study, we characterized the prepro-GnRH from the Zhikong scallop. Similar to the known GnRH precursors, the scallop GnRH precursor exhibited a conserved structure consisting of a signal peptide, a mature GnRH peptide and a cleavage site. In the comparison of all the examined species, the site of prepro-GnRH was found to be strictly conserved at the pyroglutamic N-terminal and the amidated C-terminal. In contrast to vertebrate GnRHs with 10 amino acids, invertebrate GnRHs are more variable in their length. The molluscan GnRHs exhibit the typical extra dipeptide insertion between the N-terminal pyro-Glu and His (Minakata and Tsutsui, 2016; Kim et al., 2017; Li et al., 2020). Our results clearly demonstrate that the scallop GnRH is a member of the molluscan GnRH group, and its characterization helps to clarify the structural significance of invertebrate GnRHs. By comparing the GnRHs in four species of scallops (Zhikong scallop, Yesso scallop, King scallop and Bay scallop, we found these GnRHs showed high similarity and shared a same mature GnRH peptide (Supplementary Figure S2). However, whether these scallop GnRHs has the same function in gonadal development in other species of scallops needs studying further.

In previous study, *GnRH* was more strongly expressed in ganglia than other tissues in Yesso scallop (Zhang et al., 2020), and PVG is the major producing organ rather than CPG (Nagasawa et al., 2015). Similarly, our study suggested that *GnRH* was mainly expressed in PVG rather than CPG in Zhikong scallop. We also found that the female scallops had higher expression of GnRH than the male scallops, it was also similar to the expression characteristics of Yesso scallop (Zhang et al., 2020). According to the *ISH* result, we found the GnRH neurons were mainly distributed in posterior and lateral lobes. The tissue distribution and spatial expression both showed similar characterization with those of Yesso scallop (Nagasawa et al., 2015; Zhang et al., 2020). Previous neuro-endocrine studies in snail suggested that clusters of neurons in different lobes may be involved in different reproduction process (Koene, 2010). Combined with our study, we suppose PL and LL of PVG may be the regions project into reproduction in Zhikong scallop.

In vertebrates, GnRH plays a key role in HPG axis. But research on GnRH signaling pathway is scarce in mollusks. Although vertebrate sex steroids have been detected in molluscan tissues and the sex steroid levels have seasonal changes during the reproductive cycle and seem to regulate reproductive events in mollusks (Siah et al., 2002; Avila-Poveda et al., 2015), a controversy exists regarding whether mollusks can synthesize vertebrate sex steroids (Fernandes et al., 2011; Nuurai et al., 2020; Fodor and Pirger, 2022). In Yesso scallop, a putative pathway for sex steroid synthesis has been proposed (Thitiphuree et al., 2019), and GnRH may participate in the regulation of steroidogenesis (Zhang et al., 2020). These studies make it more convincible that scallops can produce sex steroids endogenously by a potential GnRH signaling pathway. In previous study, sex steroids have been also found in Zhikong scallop and correlated with gametogenesis (Liu et al., 2014b; Zheng et al., 2014). In our study, we found *GnRH* displayed higher expression in the female than the male. *GnRH* showed significant high expression at the growing stage in PVG during the gonadal development. We suggested that the GnRH may regulate gonadal development in the growing stage of the female scallops. However, our knowledge on the GnRH function of Zhikong scallop is still fragmentary, and whether the GnRH could regulate gonadal development and/or sex steroids synthesis remains to be answered.

## Data Availability

The original contributions presented in the study are included in the article/Supplementary Material, further inquiries can be directed to the corresponding authors.
