# Supplementary material for: Identification and characterization of gonadotropin-releasing hormone (GnRH) in Zhikong scallop Chlamys farreri during gonadal development
Source: Front Physiol. 2023 May 31;14:1180725. doi: 10.3389/fphys.2023.1180725 (PMC10264684; doi:10.3389/fphys.2023.1180725)
Supplement: Supplementary file 1 [file DataSheet1.docx]

Identification and characterization of gonadotropin-releasing hormone (GnRH) in Zhikong scallop *Chlamys farreri* during gonadal development

**Juyan Tang^1^, Mengqiang Yuan^1^, Jia Wang^2^, Qianqian Li^1^, Baoyu Huang^1^, Lei Wei^1^, Yaqiong Liu^1^, Yijing Han^1^, Xuekai Zhang^1^, Xiaona Wang^1^, Meiwei Zhang^1^*, Xiaotong Wang^1^***

1. School of Agriculture, Ludong University, Yantai 264025, China

2. College of Animal Science and Technology, Northwest A&F University, Xianyang 712100, China

***Correspondence:**

meiwzh@163.com

wangxiaotong999@163.com

**Table 1.** List of primers used for ORF cloning, *in situ* hybridization and RT-qPCR analysis

| **Primer name** | **Sequence (5’–3’)** | **Application** |
| --- | --- | --- |
| GnRH_clone | F: TGATTGAACAACAGGTAGCACACAT | ORF cloning |
|  | R: TGTTATGTCGTCAGTTTGTGCGTAT |  |
| GnRH_ISH | F: ATGTCATCCTACACACAGACCCTAAT | *in situ* hybridization |
|  | R: TTACTTTGAGTCACTTGACATGCCT |  |
|  | F6: ATTTAGGTGACACTATAGATGTCATCCTACACACAGACCCTAAT |  |
|  | R7: TAATACGACTCACTATAGGGTTACTTTGAGTCACTTGACATGCCT |  |
| GnRH | F: CAAGTGACTCAAAGTAAAAAGACGG | RT-qPCR |
|  | R: TCCTGACTCTTACAAGGTTATCCAC |  |
| EF1A | F: GCCATACCGCTCACATTGCT | RT-qPCR |
|  | R: CCAGAACGACGGTCGAGTTT |  |


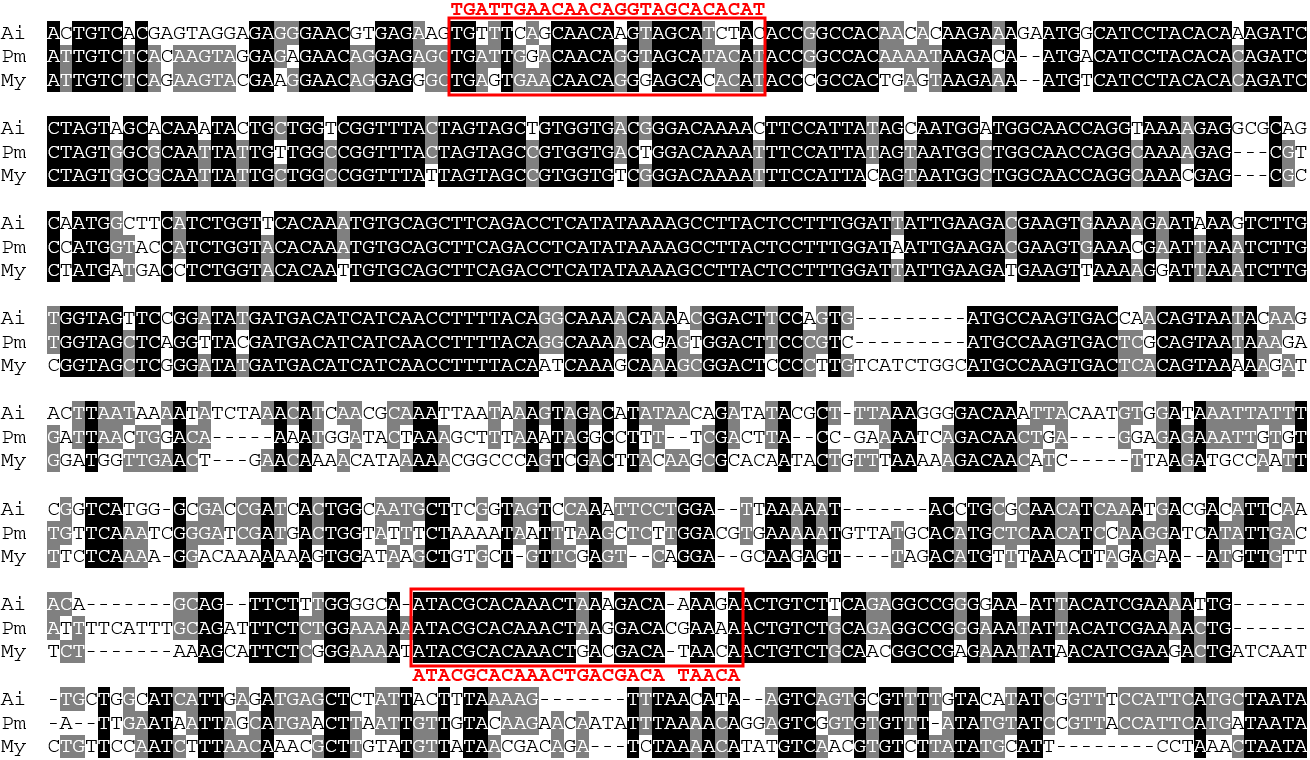
**Figure 1.** Alignment of *GnRH* nucleotide sequences. The red boxes show the designed sites of the clone primers. *Abbreviations*: Ai, *Argopecten irradians*; Pm, *Pecten maximus*; My, *Mizuhopecten (Patinopecten) yessoensis*.


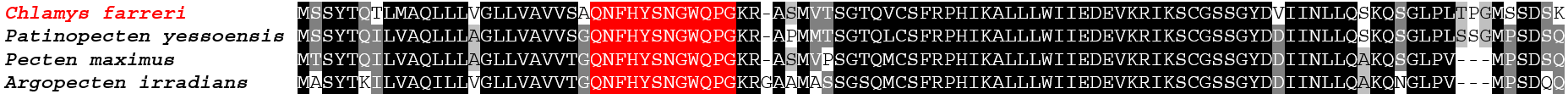


**Figure 2.** Alignment of GnRH peptides among four species of scallops. The mature GnRH peptide are highlighted in red.

**Reagent formulation for** ***in situ* hybridization**

PBST: phosphate-buffered saline plus 0.1% Tween-20

hybridization buffer: 50% formamide, 5× SSC, 100 mg/ml yeast tRNA, 1.5% blocking reagent, 0.1% Tween-20

maleic acid buffer: 0.1 M maleic acid, 0.15 M NaCl, 0.1% Tween-20, pH = 7.5
